# Annexin A2 promotes phagophore assembly by enhancing Atg16L^+^ vesicle biogenesis and homotypic fusion

**DOI:** 10.1038/ncomms6856

**Published:** 2015-01-19

**Authors:** Kateryna Morozova, Sunandini Sidhar, Valerio Zolla, Cristina C. Clement, Brian Scharf, Zoe Verzani, Antonio Diaz, Jorge N. Larocca, Katherine A. Hajjar, Ana Maria Cuervo, Laura Santambrogio

**Affiliations:** 1 Department of Pathology, Albert Einstein College of Medicine, 1300 Morris Park Avenue, Bronx, New York, New York 10461, USA; 2 Department of Developmental Molecular Biology, Albert Einstein College of Medicine, 1300 Morris Park Avenue, Bronx, New York, New York 10461, USA; 3 Department of Cell and Developmental Biology, Weill Cornell Medical College, 1300 York Avenue, New York, New York 10065, USA; 4 Department of Neurology, Albert Einstein College of Medicine, 1300 Morris Park Avenue, Bronx, New York, New York 10461, USA; 5 Department of Pediatrics, Weill Cornell Medical College, 1300 York Avenue, New York, New York 10065, USA; 6 Department of Microbiology and Immunology, Albert Einstein College of Medicine, 1300 Morris Park Avenue, Bronx, New York, New York 10461, USA

## Abstract

Plasma membrane budding of Atg-16L-positive vesicles represents a very early event in the generation of the phagophore and in the process of macroautophagy. Here we show that the membrane curvature-inducing protein annexin A2 contributes to the formation of these vesicles and their fusion to form phagophores. Ultrastructural, proteomic and FACS analyses of Atg16L-positive vesicles reveal that 30% of Atg16L-positive vesicles are also annexin A2-positive. Lipidomic analysis of annexin A2-deficient mouse cells indicates that this protein plays a role in recruiting phosphatidylserine and phosphatidylinositides to Atg16L-positive vesicles. Absence of annexin A2 reduces both vesicle formation and homotypic Atg16L vesicle fusion. Ultimately, a reduction in LC3 flux and dampening of macroautophagy are observed in dendritic cells from *Anxa2*^*−/−*^ mice. Together, our analyses highlight the importance of annexin A2 in vesiculation of a population of Atg16L-positive structures from the plasma membrane, and in their homotypic fusion to form phagophore structures.

## Abstract (web summary)

The earliest steps in autophagy are thought to include the budding of Atg16L-containing vesicles from the plasma membrane and their homotypic fusion to form a phagophore. Morozova *et al*. reveal a role for the membrane curvature-inducing protein Annexin A2 in the formation and fusion of these vesicles.

## Graphical abstract

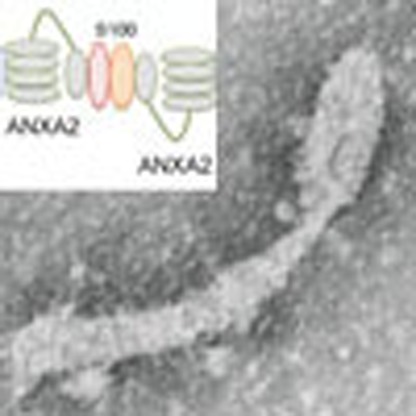

Macroautophagy (MA) is a basic catabolic pathway that is highly conserved in eukaryotes and is used by cells for the delivery of membrane-encapsulated cellular cargos to endosomes/lysosomes for degradation^1^,^2^. The process of encapsulation of organelles and biomolecules is initiated in the cytosol by the formation of a double-membrane structure called the phagophore^3^. The membrane sources that give rise to the phagophore include the endoplasmic reticulum, Golgi, mitochondria and plasma membrane^4^,^5^,^6^,^7^,^8^,^9^,^10^,^11^,^12^,^13^. Indeed, it is becoming increasingly apparent that the phagophore may originate differently during physiologic or stress-related conditions. Recruitment of lipids and proteins from different membrane sources increases the basal level of MA and satisfies the requirement to dispose of cellular structures in different locations^4^,^5^,^6^,^7^,^8^,^9^,^10^,^11^,^12^,^13^. In addition, different cell types may develop phagophores by preferential routes according to their specific autophagic requirements^4^,^5^,^6^,^7^,^8^,^9^,^10^,^11^,^12^,^13^.

For phagophores that originate from the plasma membrane, it has been proposed that Atg-16L-positive vesicles directly bud from the plasma membrane, and generate pre-phagophore structures either by direct homotypic fusion of vesicles, or after trafficking to early/recycling endosomes, by heterotypic fusion with Atg9^+^ vesicles^2^,^4^,^5^. Subsequent pairing of Atg16L with the conjugated protein pair Atg12-Atg5 results in the generation of tubulovesicular double membranes, which elongate further to form the autophagosome^7^. Coincident with the arrival of Atg12/5 to the phagophore, Atg16L-positive compartments also acquire LC3, which gives rise to the mature phagophore. Once the elongation is complete, the double membranes seal to complete the autophagosome; Atg12, Atg5 and Atg16L are then released into the cytosol, whereas part of LC3 remains associated with the autophagosome until either its fusion with lysosomes to become an autolysosome, or its fusion with late endosomes to form an amphisome.

The machinery regulating Atg16L vesicle homotypic fusion was recently found to comprise the SNARE protein VAMP7 and its partner SNAREs (syntaxin 7, syntaxin 8 and Vti1b)^7^. On the other hand, heterotypic fusion of Atg16L-Atg9 vesicles relies on the SNARE protein VAMP3 (ref. 4). Both Atg16L^+^ and Atg9^+^ vesicles have been observed in Rab11^+^-recycling endosomes, where the fusion events are likely to occur^4^,^5^.

Annexin A2 is a cytosolic soluble protein, present in most eukaryotic cells, that binds membrane phospholipids in presence of Ca^2+^. Annexin A2 has been associated with multiple intracellular functions, including vesicle budding, fusion, internalization, late endosomal biogenesis and membrane repair^14^,^15^,^16^,^17^,^18^. At the plasma membrane, annexin A2 has been shown to induce the formation of phosphatidylinositol 4,5-bisphosphate (PI(4,5)P2)-rich domains, which are important for the local inward curvature of the cell’s lipid bilayer^19^,^20^. In ddition, annexin A2 has been shown to be pivotal for the biogenesis of the late endosomal multivesicular bodies^21^,^22^. Thus, annexin A2 enables membrane lipid segregation, membrane deformation and associated inward vesiculation^23^,^24^,^25^.

A recent analysis performed in our laboratory on annexin A2^+^-purified vesicles^26^ indicated that some of these vesicles were Atg16L^+^. In the present study, we investigated the role of annexin A2 in Atg16L^+^ vesicle formation, and its overall participation in MA. Our data indicate that annexin A2 aids the vesiculation of Atg16L-positive vesicles at the plasma membrane by recruiting phosphatidylserine (PS) and phosphatidylinositides, and by promoting Atg16L-mediated fusion to form phagophore structures in early MA.

## Results

### Annexin A2 associates with Atg16L^+^ vesicles

**Figure 1:**
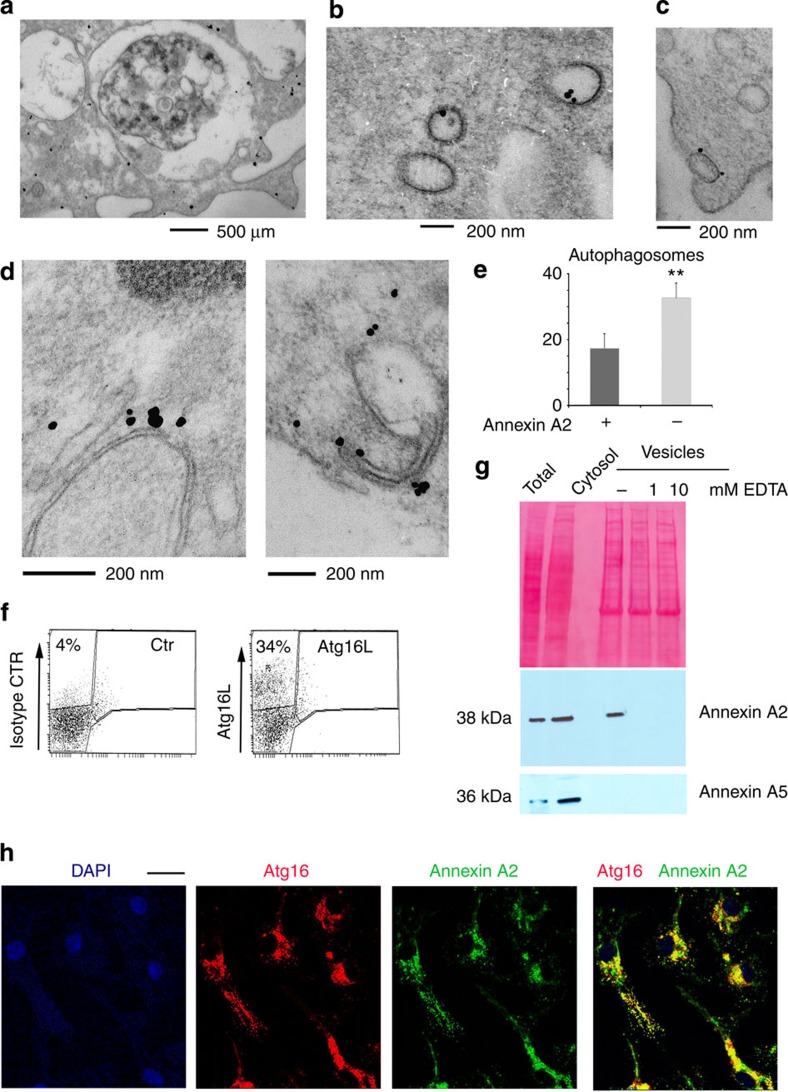
DC phagophore precursors and autophagosome are annexin A2-positive. (**a**–**c**) Immunogold staining of dendritic cells with annexin A2 antibody identifies positive staining in the (**a**) cytosol, (**a**) plasma membrane, (**a**) multivesicular late endosomes (MVB) and (**b**,**c**) vesicular structures: *n*≥50 cells. (**d**) Immunogold labelling of phagophore structures with annexin A2 antibody; *n*≥50 cells. (**e**) Quantification of annexin A2-positive and -negative autophagosomes in primary dendritic cells; the mean and s.e. were calculated from *n*≥50 cells: *P*<0.01 (unpaired two-tailed Student’s *t*-test). (**f**) FACS analysis of total DC vesicles prepared by cytosol ultracentrifugation. Vesicles were stained with an Atg16L antibody that detects ~30% of positively labelled vesicles. Shown is a single representative sort (*n*=14 sorts). (**g**) Representative western blot analysis of Atg16L-positive vesicles, FACS-sorted as in **f**, for annexin A2 and annexin A5. Ponceau staining is shown as loading control (*n*=3 blots). (**h**) Immunofluorescence of primary dendritic cells stained with anti-annexin A2 and anti-Atg16L antibodies; *n*≥75 cells. Scale bar: 10 μm.

In agreement with the reported biological roles of annexin A2, ultrastractural analysis of immunogold-stained, primary mouse dendritic cells showed the presence of annexin A2 at the plasma membrane, in the cytoplasm, bound to vesicles of variable size and in late endosomal compartments (Fig. 1a–c). Annexin A2 staining was also observed on phagophore-like structures present in the cytosol (Fig. 1d), on ~20% of autophagosomes (Fig. 1e), and on vesicles budding from the plasma membrane (Fig. 1c), reflecting results reported previously reported for Atg16L^+^ vesicles involved in phagophore formation^7^. Thus, we asked whether both proteins could be involved in cellular events that provide membrane for phagophore formation in the initial stages of MA.

Total cellular vesicles were prepared using ultracentrifugation of the cellular cytosol from the mouse dendritic cell line JAWS II (DC). Pelleted vesicles were stained with a monoclonal antibody to Atg16L, or an isotype control, and sorted using flow cytometry to specifically separate Atg16L^+^ vesicles from the total vesicle population (Fig. 1f). Vesicle lysates were resolved using SDS–PAGE and immunoblotted for annexin A2, with total cell lysates and cytosol included as positive controls (Fig. 1g, Supplementary Fig. 1). To establish the Ca^2+^ dependence of annexin A2 association with vesicular structures, the vesicles were left untreated or incubated with EDTA before lysis. Western blot analysis revealed the presence of annexin A2, but not annexin A5, in Atg16L-positive vesicles, and confirmed the role of Ca^2+^ in recruitment of annexin A2 from the cytosol (Fig. 1g, Supplementary Fig. 1). Colocalization between Atg16L and annexin A2 was further confirmed using immunofluorescence (Fig. 1h).

### Proteomic analysis of Atg16L/annexin A2^+^ vesicles

**Figure 2:**
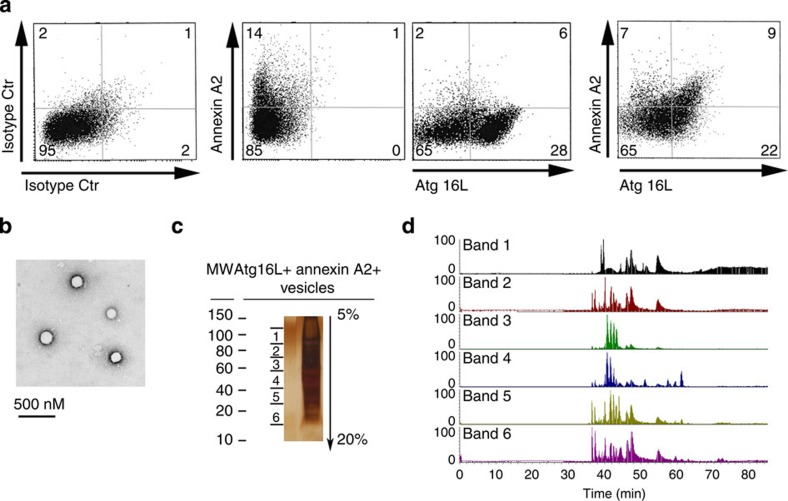
Proteomic characterization of Annexin A2^+^/Atg16^+^ vesicles. (**a**) FACS analysis of annexin A2^+^ and Atg16L^+^ vesicles (*n*=14 sorts). (**b**) Ultrastructural analysis of annexin A2^+^/Atg16L^+^-sorted vesicles. (**c**) Silver staining of an SDS–PAGE gel loaded with lysates of annexin A2^+^/Atg16L^+^-sorted vesicles. The gel was divided into six bands for mass spectrometric analysis. (**d**) Chromatogram of each gel band following protein extraction and trypsin digestion. Proteins identified by mass spectrometric analysis are listed in Table 1. Data presented in **c**,**d** are representative of three separate experiments.

Next, we investigated the protein composition of annexin A2^+^/Atg16L^+^ vesicles to further define their role in MA. Total cellular vesicles were prepared from isolated DC cytosol. Following staining with Atg16L and annexin A2 antibodies, doubly positive vesicles were sorted (Fig. 2a). Quantitative FACS analysis indicated that 28% of the total vesicles were Atg16L^+^. These data are consistent with the notion that, unlike other cell types, autophagy is highly active in dendritic cells, even under physiological conditions due to their role in immunosurveillance, which requires constant transport of the extra- and intracellular proteome to endo/lysosomal compartments^27^,^28^,^29^. In addition, dendritic cells, as opposed to other cell types, are highly phagocytic. Thus, it is likely that many of the vesicles that give rise to phagophores derive from the plasma membrane in this cell type. Of the total Atg16L^+^ vesicles, ~30% were also annexin A2^+^, indicating that the combined markers define a subpopulation of autophagic precursors (Fig. 2a). Likewise, only about half of annexin A2^+^ vesicles were also Atg16L^+^ (Fig. 2a), consistent with the notion that annexin A2 is implicated in other cellular functions besides autophagy. The purity of the sorted vesicles was confirmed with ultrastructural morphology (Fig. 2b).

To further analyse the origin of the vesicles, sorted material was lysed and proteins resolved with SDS–PAGE. Excised bands were digested with trypsin and the retrieved peptides analysed using mass spectrometry (Fig. 2c,d, Table 1). Several proteins known to participate in vesicle formation at the plasma membrane were identified; these included clathrin, the AP-2 adaptor complex, and accessory proteins such as synaptotagmin II, an AP2 binder, and endophilin A3, a protein involved in vesicle fission. Cytoskeletal proteins, known to be involved in vesicle rocketing and movement, were also detected; these included actin, dynactin and cofilin 1 (ref. 24). In addition, the SNARE protein VAMP7 and its binding partners Vti1b, Hrb and ARF 6 were also identified^7^. These SNAREs were previously shown to be associated with Atg16^+^ vesicles (Table 1)^7^. VAMP3, the SNARE involved in heterotypic Atg16L-Atg9 vesicle fusion, was also detected by proteomic analysis^5^. Girdin and Aurora kinase A, inhibitors of LC-3 vesicle binding, were also sequenced^30^,^31^, as well as several proteins involved in phosphatidylinositol (PI) metabolism including, synaptojanin 2, an enzyme that dephosphorylates PI at positions 3, 4, 5 of the inositol ring to form PIP2. Finally, Vps13, a vacuolar sorting protein required for efficient phagocytosis, membrane bending and PI–phosphate regulation, was also detected^32^,^33^ (Table 1). Together, the results clearly validate that the Atg16^+^-annexin A2^+^ vesicle subpopulation originates from the plasma membrane and contains multiple molecular effectors involved in vesicle docking and fusion.

### Annexin A2 generates PI- and PS-enriched Atg16L^+^ vesicles

**Figure 3:**
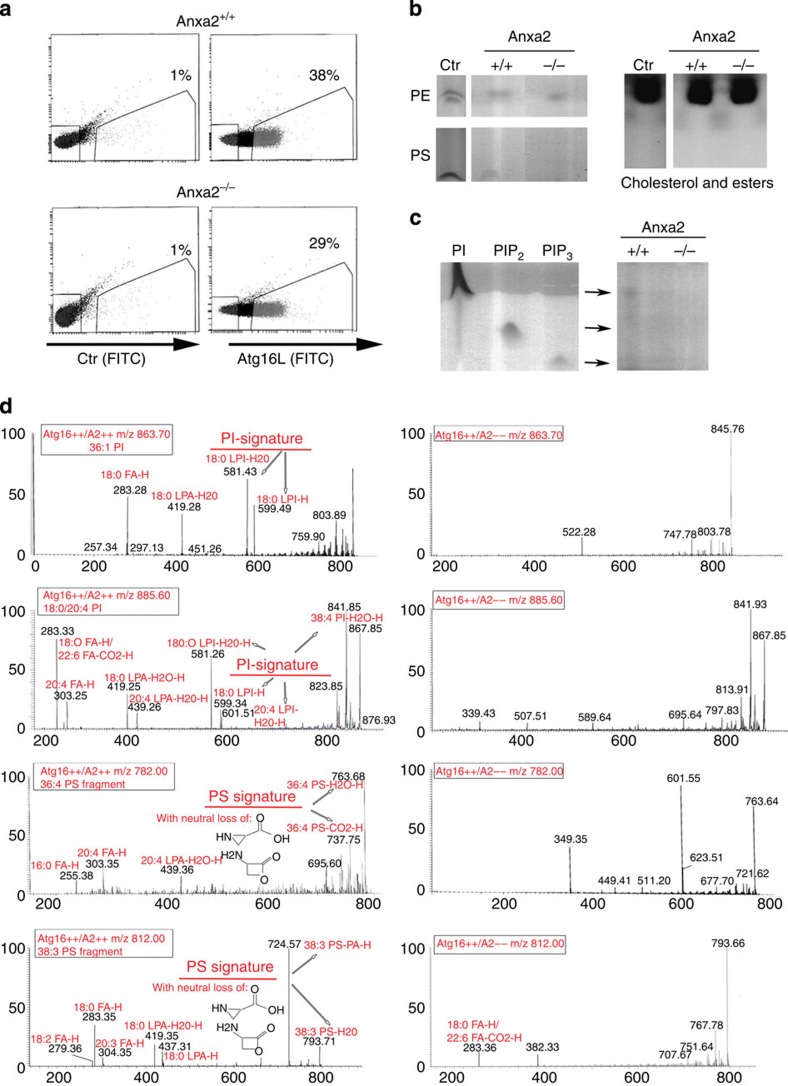
Atg16L^+^ vesicles from *Anxa2*^*−/−*^ mice are deficient in PI and PS. (**a**) FACS analysis of Atg16L^+^ vesicles in primary dendritic cells from *Anxa2*^*+/+*^ and *Anxa2*^*−/−*^ mice. (**b**) Thin layer chromatography of cholesterol, PE and PS extracted from Atg16L^+^ vesicles sorted from dendritic cells of *Anxa2*^*+/+*^ and *Anxa2*^*−/−*^ mice. (**c**) Thin layer chromatography of PI, PIP2 and PIP3 extracted from Atg16L^+^ vesicles sorted from *Anxa2*^*+/+*^ and *Anxa2*^*−/−*^ dendritic cells. Data presented in **a**,**b** are representative of four separate experiments. (**d**) Mass spectrometry of lipids extracted from Atg16L^+^ vesicles sorted from *Anxa2*^*+/+*^ and *Anxa2*^*−/−*^ dendritic cells; data are representative of four separate experiments.

At the plasma membrane, annexin A2 facilitates the formation of lipid microdomains enriched in the phosphoinositide PI (4,5)P2, which promotes membrane deformation and actin-mediated vesicle rocketing^19^,^23^. Conversely, annexin A2 binds PS to facilitate endosomal biogenesis or membrane repair in both endosomes and at the plasma membrane^21^,^26^. To determine whether the formation of Atg16L^+^-annexin A2^+^ vesicles is supported by PS and/or PI recruitment, Atg16L^+^ vesicles were sorted from the cytosol of bone marrow dendritic cells (BMDCs) from wild-type (*Anxa2*^*+/+*^) and annexin A2 knockout (*Anxa2*^*−/−*^) mice (Fig. 3a). Total lipids were extracted from 1 × 10^5^ vesicles and separated using thin layer chromatography. Purified lipids were used as controls (Fig. 3b). No differences were observed between *Anxa2*^*+/+*^ and *Anxa2*^*−/−*^ vesicles with respect to cholesterol and cholesterol esters, the most abundant vesicular lipids (Fig. 3b). In addition, no apparent differences were observed in the abundance of PE, the lipid required for LC3 recruitment to the phagophore, between vesicles from the two genotypes. On the other hand, a quantitative decrease in recovered PS was observed in *Anxa2*^*−/−*^ versus *Anxa2*^+/+^ vesicles. Of the three analysed phosphatides (PI, PIP2 and PIP3), only trace amounts of PI were detected in *Anxa2*^*+/+*^, but not *Anxa2*^*−/−*^, vesicles (Fig. 3c); this result is not surprising considering the low sensitivity of thin layer chromatography and the very low abundance of each of these signalling lipids in membrane structures.

To further analyse the lipid content of *Anxa2*^*+/+*^ and *Anxa2*^*−/−*^ vesicles, we performed mass spectrometry (MS/MS) analysis on purified vesicle-derived lipids (Fig. 3d, Supplementary Data 1). MS/MS revealed that both PA and PE were present in vesicle lipids from both sources, whereas neither PS nor PI was detected in *Anxa2*^*−/−*^ vesicles (Supplementary Data 1, Fig. 3). Using MS, we were able to detect neither PIP2 nor PIP3, both of which are known to be present in trace amounts. Thus, even though it is possible that both lipids are present in the vesicles, they are probably found in very low concentrations, below our level of experimental detection. Together, these data support the requirement for annexin A2 in plasma membrane vesiculation of Atg16L^+^ vesicles, and its role in forming PI–PS-enriched microdomains to sustain this process.

### Annexin A2 promotes homotypic fusion of Atg16L^+^ vesicles

**Figure 4:**
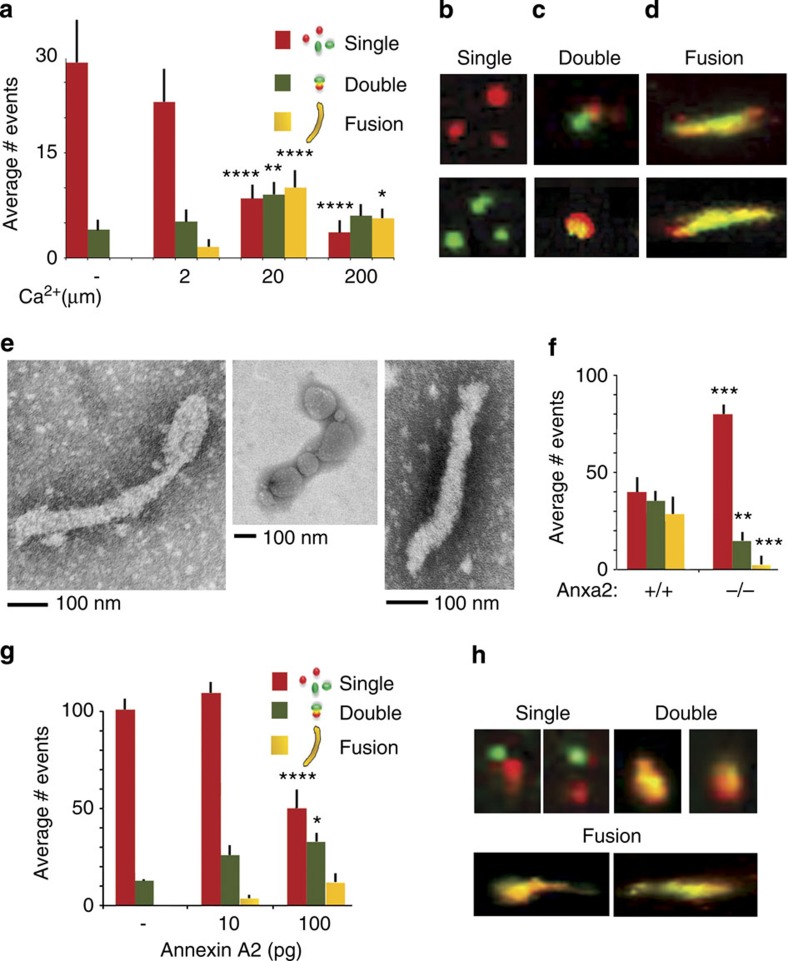
Annexin A2 is required for homotypic Atg16L^+^ vesicle fusion. (**a**–**d**) Analysis of Atg16L^+^/annexin A2^+^ vesicles fusion events at different Ca^2+^ concentrations; Atg16L^+^/annexin A2^+^ vesicles were sorted with Alexa-488 or Texas-Red-conjugated antibody to obtain mixed colour-positive vesicles. *In vitro* fusion was analysed at different Ca^2+^ concentrations. Single vesicles refer to structures as depicted in **b**; double vesicles refer to structures as depicted in **c** and multiple fusion refers to structures as depicted in **d**; the mean and s.e. were calculated from *n*>300 events. *P<0.05; **P<0.01; ****P<0.0001. (**e**) Ultrastructural analysis of phagophore-like structures derived from the *in vitro* fusion of Atg16L^+^/annexin A2^+^ vesicles. (**f**) Quantification of fusion events among Atg16L^+^ vesicles sorted from *Anxa2*^*+/+*^ and *Anxa2*^*−/−*^ DCs; the mean and s.e. were calculated from *n*>300 events. **P<0.01; ***P<0.001. (**g**,**h**) Quantification of fusion events among Atg-16L vesicles sorted from *Anxa2*^*+/+*^ and *Anxa2*^*−/−*^ DCs, plus or minus reconstitution with annexin A2; the mean and s.e. were calculated from *n*>300 events. *P<0.05; ****P<0.0001 (all unpaired two-tailed Student’s *t*-test).

Although annexin A2 is not a fusogenic protein *per se*, it has been associated with Ca^2+^-mediated membrane fusion events. In association with its binding partners (S100A10 and S100A11), annexin A2 is known to bridge disparate membranes and promote fusion^17^,^20^. We, therefore, evaluated the potential role for annexin A2 in Atg16L-mediated phagophore formation and elongation using a comparative fluorescence-based fusion assay. Atg-16L^+^ vesicles were isolated from *Anxa2*^*+/+*^ BMDC using Atg16 antibody and FACS sorting. The vesicles were labelled with either red or green fluorochromes, incubated for 30 min in fusion buffer, and equilibrated in a range of Ca^2+^ concentrations. As expected, fusogenic events were quantitatively proportional to the Ca^2+^ concentration (Fig. 4a–d). In addition, visualization of fusion products by EM confirmed the presence of elongated, double-membrane structures that possessed ultrastructural characteristics of an emerging phagophore (Fig. 4e).

Next, Atg16L^+^ vesicles isolated from *Anxa2*^*+/+*^ and *Anxa2*^*−/−*^ BMDC were subjected to *in vitro* fusion at 20 μm Ca^2+^. In immunofluorescence studies, we noted an equal presence of single and double vesiculate structures, as well as elongated fusion products, in *Anxa2*^*+/+*^ samples, whereas the majority of *Anxa2*^*−/−*^ samples contained mainly single vesicles with a dramatically reduced number of fusion structures (Fig. 4f). Fusion events could be partially restored in *Anxa2*^*−/−*^ samples upon addition of recombinant annexin A2 (Fig. 4g,h). Together, the data indicated an additional role of annexin A2 in homotypic fusion of Atg16L^+^ vesicles^7^.

### Annexin A2 is required to sustain autophagic flux

**Figure 5:**
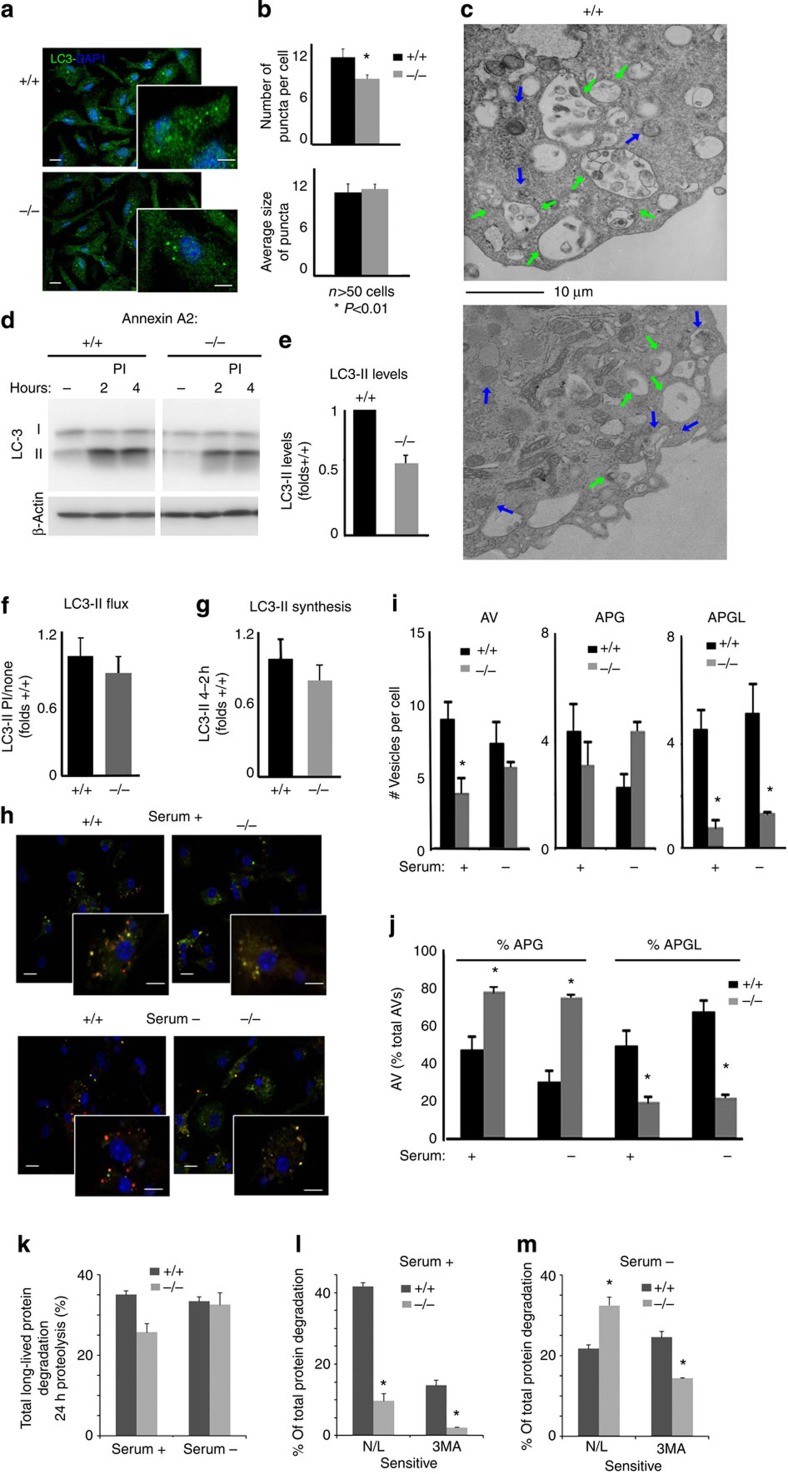
Reduced autophagosome biogenesis and autophagic flux in DC from *Anxa2*^*−*/*−*^ mice. (**a**) Immunofluorescence for LC3 in dendritic cells of *Anxa2*^*+/+*^ and *Anxa2*^*−/−*^ mice. Nuclei are highlighted with DAPI. Insert shows higher magnification. Scale bars: 10 and 5 μm (insert). (**b**) Quantification of the number of puncta per cell, and average size of LC3-positive puncta, in micrographs such as shown in **a**; the mean and s.e. were calculated from *n*>75 cells; *P<0.05. (**c**) Electron microscopy of the same cells. Arrows mark autophagosomes (blue) and autolysosomes (green). Scale bar: 10 μm. (**d**) Immunoblot for LC3 in *Anxa2*^*+/+*^ and *Anxa2*^*−/−*^ dendritic cells untreated (none) or incubated with lysosomal protease inhibitors (PI) for the indicated times. Actin is shown as loading control. (**e**–**g**) Quantification of steady-state levels of LC3-II (**e**), increase in LC3-II levels upon lysosomal proteolysis blockage (**f**) and increase in LC3-II at the two times of PI treatment (**g**) calculated from immunoblots shown in **d**. *n*=3. (**h**) Direct fluorescence microscopy in *Anxa2*^*+/+*^ and *Anxa2*^*−/−*^ dendritic cells transiently transfected with the tandem reporter mCherry-GFP-LC3, and maintained in serum-supplemented or serum-depleted medium for 6 h. Inserts show higher magnification images. Scale bars: 10 and 5 μm (insert). (**i**) Quantification of the number of total autophagic vacuoles (AV; mCherry+). Autophagosome (APG, mCherry+ and GFP+) and autolysosomes (APGL, mCherry+ and GFP−) in images such as in **h**; the mean and s.e. were calculated from *n*>75 cells; *P<0.05. (**j**) Quantification of the percentage of autophagosomes and autolysosomes; *P<0.05. (**k**–**m**) Rates of degradation of long-lived proteins in *Anxa2*^*+/+*^ and *Anxa2*^*−/−*^ dendritic cells maintained in the presence or absence of serum for 24 h. Total rates of protein degradation (**k**), and degradation sensitive to lysosomal inhibition (combination of ammonium chloride and leupeptin (N/L)) or to inhibition of macroautophagy (3-methyl adenine (3MA)), in cells maintained in the presence (**l**) or absence (**m**) of serum. *n*=6; *P<0.05 (all unpaired two-tailed Student’s *t*-test).

To analyse the consequences of annexin A2 depletion on MA, we first stained *Anxa2*^*+/+*^ and *Anxa2*^*−/−*^ dendritic cells for endogenous LC3 (Fig. 5a,b). We found a significant reduction in the number of LC3+ vesicles in *Anxa2*^*−/−*^ cells, but no change in the average size of the LC3+ puncta (Fig. 5b). Electron microscopy also confirmed the lower abundance of autophagic vacuoles in *Anxa2*^*−/−*^ cells, with no apparent change in the morphology and size of completed vesicles (Fig. 5c). These findings support the hypothesis that annexin A2 is required for the early steps of autophagosome biogenesis (nucleation); however, it is not a determinant of final autophagosome size, in which Atg-16L fusogenicity is less important.

The reduced content of autophagic vacuoles in *Anxa2*^*−/−*^ dendritic cells could reflect either slower formation or accelerated lysosomal degradation of the formed vesicles. Direct analysis of autophagic flux, assessed by measuring LC3 degradation (Fig. 5d–g), and maturation of autophagic compartments, highlighted by the tandem reporter mCherry-GFP-LC3 (Fig. 5h–j), suggests that depletion of annexin A2 leads to reduced autophagic flux. Immunoblot analysis revealed lower steady-state levels of LC3-II (Fig. 5d,e, Supplementary Fig. 1), and reduced accumulation of LC3-II upon treatment with inhibitors of lysosomal proteolysis in *Anxa2*^*−/−*^ versus *Anxa2*^*+/+*^ cells (Fig. 5d,f). Analysis of autophagosome biogenesis, assessed as the increase in LC3-II at two times during proteolysis inhibition, suggests that the reduced autophagic flux in *Anxa2*^*−/−*^ cells is due mainly to reduced autophagosome formation (Fig. 5i,j). We also used the tandem reporter mCherry-GFP-LC3, which allows for a more dynamic measurement of both autophagosome formation and maturation; acidification of autophagosomes upon lysosomal fusion quenches their green fluorescent protein (GFP) fluorescence, whereas mCherry fluorescence persists, allowing for identification of autolysosomes as red-only puncta. Direct fluorescence confirmed that *Anxa2*^*−/−*^ cells maintained in serum-containing medium had a reduced number of both autophagosomes and autolysosomes (Fig. 5h,j). In agreement with these morphological data, *Anxa2*^*−/−*^ cells maintained under these conditions also showed a significant decrease in the total degradation rates of long-lived proteins (Fig. 5k) that was even more pronounced when we analysed the fraction of proteins degraded in the lysosomal compartment, as evidence by their sensitivity to inhibition of lysosomal proteolysis, or those whose degradation was directly dependent on active autophagy, as evidenced by their sensitivity to the inhibition of autophagosome biogenesis by the PI3K inhibitor 3-methyladenine (Fig. 5l). Interestingly, the reduced autophagosome content in *Anxa2*^*−/−*^ cells was no longer observed upon induction of autophagy by starvation (Fig. 5h,i). These data suggest that the contribution of the plasma membrane, and consequently the involvement of annexin A2 in early precursor fusion, is more important for basal, quality-control autophagy than for induced autophagy.

Lastly, analysis of the compartments highlighted by the tandem LC3 reporter revealed that, in addition to the reduced number of autophagic vacuoles in the *Anxa2*^*−/−*^ cells, there was a higher proportion of autophagosomes than autolysosomes in these cells (Fig. 5h,j). The slower maturation of autophagic compartments in cells defective in annexin A2 was also confirmed by electron microscopy analysis (Fig. 5c). In contrast to the advanced degradation of cargo in most of autophagic vacuoles in *Anxa2*^*+/+*^ cells (compatible with post-lysosomal fusion compartments), cargo was still clearly distinguishable inside autophagic vacuoles (pre-lysosomal fusion) in the *Anxa2*^*−/−*^ cells. In fact, this defective maturation was still observed upon serum removal, despite the fact that autophagosome biogenesis was partially restored under these conditions (Fig. 5i,j). In agreement with these findings, the fraction of long-lived proteins degraded by MA (sensitive to 3MA) upon serum removal was still significantly lower in *Anxa2*^*−/−*^ cells (Fig. 5m). Interestingly, despite reduced autophagic degradation in *Anxa2*^*−/−*^ cells during serum deprivation, total degradation rates of long-lived protein were comparable in both cell groups under these conditions (Fig. 5k). This sustained protein degradation is likely due to compensatory upregulation of other autophagic pathways, because the fraction of lysosomal degradation persistent upon inhibition of MA was elevated in these cells upon serum removal (Fig. 5m). It is possible that the differences in lipid composition (PS and PI) in autophagosomes formed in the absence of annexin A2 determine their lower fusogenic capability with respect to endosomal and lysosomal compartments.

Overall, our findings reveal that annexin A2 is required for the homotypic fusion of Atg16L+ vesicles in the formation of pre-autophagosome structures, and suggest that proper recruitment of phospholipids to the forming autophagosome membrane is required for the later maturation of the autophagic compartments.

## Discussion

During the last few years it has become apparent that the phagophore may arise from multiple membranes, including the ER–Golgi, mitochondria and plasma membrane^1^,^4^,^7^,^9^,^10^,^7^,^8^,^9^,^10^,^13^. It is possible that in different cell types, the phagophore originates preferentially from different subcellular locations, or that, even within the same cell, the site of origin can change according to the nature of the stressor that induces MA. The plasma membrane origin of the phagophore relies on Atg16L-positive vesicles, which bud inwardly and give rise to phagophores through homotypic fusion or heterotypic fusion with Atg9^+^ vesicles after trafficking to the recycling endosomes^4^,^5^,^7^. SNARE proteins involved in homotypic fusion have been identified as VAMP7, and its partners, ARF6 and Vtib, whereas the SNARE that controls heterotypic Atg16^+^–Atg9^+^ fusion is VAMP3 (ref. 4, 7).

Formation of the phagophore from the Golgi membrane requires generation of PI(3)P, from PI, through the action of the class III phosphoinositide kinase (PI3K-III) Vps34 (ref. 1). In addition, a PI3K-III-independent mechanism, which generates PI(3-4)P2 and relies on PI3K-II, has also been described^34^. Alternative sources of PI(3)P formation are dephosphorylation of PI(3-4)P2 and PI(3-5)P2 by PI4 or PI5 phosphatases, respectively^35^,^36^. At the plasma membrane, on the other hand, Atg16L interacts with the clathrin heavy chain and ARF6, and promotes autophagosome formation through activation of PIP5 kinase, thus generating PI(4-5)P2. Both ARF6 knockdown and impaired generation of PI(4-5)P2 decrease formation of pre-phagophore structures^6^,^7^.

Annexin A2 is a ubiquitously expressed, soluble protein that has been implicated in multiple biological processes related to membrane trafficking and fusion^17^. Our analyses of the early events involved in MA identified two additional functions for annexin A2. First, annexin A2 participates in the generation of PI–PS-enriched Atg16L-positive vesicles. It was previously shown in liposome-based assays that annexin A2 promotes the recruitment of PI(4,5)P2, cholesterol and glycosphingolipids into liposomes, and also promotes membrane indentations at sites rich in PI(4,5)P2, leading to inward membrane budding and vesiculation^19^,^20^,^23^. These functions can be performed by the full-length protein, as well as by the C-terminal protein core domain. Annexin A2 has also been shown to recruit PS at both the plasma and endosomal membrane, and it has been proposed that annexin A2 has a scaffolding function in the biogenesis of membrane structures. In particular, by organizing lipid microdomains, annexin A2 regulates membrane curvature, providing a driving force for budding^37^.

Through proteomic analysis, we have discovered that a subset of annexin A2^+^ vesicles are also Atg16L^+^. Further proteomic assessment of vesicle content has identified several proteins previously known to be associated with Atg16L^+^ phagophore precursors, thus establishing the plasma membrane as the site of vesicular origin. Lipidomic analyses have demonstrated, furthermore, that annexin A2 is required for PS recruitment in Atg16L^+^ vesicles from the plasma membrane. Although we could not directly demonstrate the presence of PI (4,5)P2 in the Atg16L vesicles, it is clear that annexin A2 is required for enrichment of vesicles with the PI (4,5)P2 precursor, PI.

The importance of PI and PS enrichment in Atg16L^+^ vesiculation is highlighted by the 30% reduction in the proportion of Atg 16L vesicles in *Anxa2*^*−/−*^ dendritic cell cytosol. Phospholipid enrichment and associated vesiculation are generated by the ability of annexin A2 to bind PS and PI in a Ca^2+^-dependent manner, and also by the ability of annexin A2 to bind and organize cytoskeleton proteins for inward budding. Indeed, annexin A2 can bind the barbed ends of F-actin filaments and generate vesicle rocketing^23^. Annexin A2-mediated vesiculation is likely aided by other proteins, such as WASP, which induces actin assembly at the surface of endomembranes, because the WASP-binding partner WIPF1 was identified in our proteomic analysis. Together, our data indicate that annexin A2 generates PS and PI microdomains, and promotes Atg16L^+^ vesiculation. It remains to be established whether the remaining Atg16L^+^ vesicles originate from other subcellular compartments in an annexin A2-independent manner.

A second annexin A2 function, reported here, is to facilitate homotypic Atg16L^+^ vesicle fusion, which is required for phagophore formation and elongation. Our *in vitro* assay demonstrates that vesicles lacking annexin A2 fuse less, and the autophagosomes that do form display further fusion defects when they encounter endosomes and lysosomes. Importantly, this decreased fusion capacity translates *in vivo* into a decrease in dendritic cell autophagic flux as determined by a reduction in LC3 processing.

In Atg16L^+^ vesicle fusion events, annexin A2 likely serves to bridge disparate vesicles. This role is well characterized for the heterotetrameric complex formed by the association of two molecules of annexin A2 and two of an S100 protein, either S100A10 or S100A11. During the bridging event, each molecule of annexin A2 engages vesicular PS, or other anionic phospholipid, electrostatically, whereas S100A10/11 proteins serve to stabilize the annexin A2 dimer. This function is mediated by the core domain of annexin A2 and requires Ca^2+^ (ref. 17).

It is important to note that our model was developed in primary dendritic cells. Compared with other cell types, dendritic cells have the highest rate of plasma membrane turnover, due to their incessant endocytic activity, which is tightly connected to their role in immunosurveillance. Thus, in this cell type it is possible that the contribution of plasma membrane to the origin of phagophore precursors is greater than in other cell types. Indeed, among the total vesicle population prepared from dendritic cell cytosol, a large proportion was Atg16L^+^, indicating a cellular commitment to autophagic processes.

In conclusion, our data support the concept that annexin A2 plays a central role in the formation and fusion of Atg16L^+^ vesicles by orchestrating recruitment of phosphoinositides and PS to vesicular membranes, and by coordinating vesicular budding and homotypic fusion. Our data elucidate a key early mechanistic step in MA.

## Methods

### Mice and bone marrow cultures

Female, *Anxa2*^*+/+*^and *Anxa2*^*−/−*^ mice on the C57Bl6 background, 8–12 weeks old, were maintained in animal facilities at Weill Cornell Medical College and Albert Einstein College of Medicine. Animal killing and bone marrow harvesting were carried out according to protocols approved by the Animal Institute Committee of both institutions. To obtain BMDCs, the bone marrow was harvested from the femurs and cells were cultured for 7 days in granulocyte–monocyte colony-stimulating factor (10 ng ml^−1^) in complete DMEM^26^.

### Immunogold labelling of annexin A2

BMDCs were fixed in 3.7% paraformaldehyde 0.1% glutaraldehyde in dPBS for 20 min followed by permeabilization in 0.1% Triton X100 for 15 min at 25 °C. Cells were extensively washed with dPBS and incubated in blocking buffer (1% FBS, 1% BSA in dPBS) for 45 min. BMDCs were then incubated overnight with 4 μg ml^−l^ goat anti-mouse annexin A2 antibody (clone A-15, Santa Cruz Biotechnology) at 4 °C, and subsequently with 0.8 μg ml^−1^ of ultrasmall gold-conjugated rabbit anti-goat IgG (Electron Microscopy Sciences, Hatfield, PA, USA). Silver enhancement of ultrasmall conjugates was performed using the Aurion R-Gent SE-EM kit (Electron Microscopy Sciences). The cells were briefly post-fixed in 1.0% aqueous osmium tetroxide and processed for transmission electron microscopy (TEM).

### Transmission electron microscopy

Primary BMDCs were fixed in 2.5% glutaraldehyde, 2% paraformaldehyde in sodium cacodylate buffer 0.1 M, pH 7.4 for 3 h at 4 °C. Samples were post-fixed in 1.0% aqueous osmium tetroxide (pH 7.4) for 1 h at 4 °C and dehydrated in a series of water/acetone mixtures progressing to 100% acetone. Cells were infiltrated in sequentially increasing concentrations of Embed 812-Araldite (Electron Microscopy Sciences), and embedded in BEEM capsules. Ultrathin sections were stained with uranyl acetate followed by lead citrate, and viewed with a Jeol JEM-1200EX transmission electron microscope (Jeol Ltd., Akishima, Japan) at 80 kV.

### Immunofluorescence

BMDCs were cultured on 22 × 40-mm glass coverslips coated with poly-L-lysine in 60-mm Petri dishes. Samples were fixed in 4% paraformaldehyde in PBS pH 7.4 for 15 min at room temperature, permeabilized with PBS and 0.5% saponin for 10 min, and blocked in 1% BSA in PBS for 30 min. BMDCs were then incubated with annexin A2 1:100 (Clone A-15, Santa Cruz Biotechnology) and Atg16L1 1:200 (Clone AB1, Sigma or Clone 1F12, MBL), in 1% BSA in PBST overnight at 4 °C. Samples were incubated with secondary antibodies (goat anti-Alexa-488 and rabbit anti-Alexa 568) for 1 h at room temperature. Samples were mounted with ProLong Gold antifade reagent with DAPI (Invitrogen), and imaged using an Olympus IX70 inverted microscope equipped with the I.P. Lab Spectrum imaging software (Scanalytics Inc., Fairfax, VA, USA).

### Negative staining of vesicles

Before and after fusion, FACS-sorted vesicles (10 μl) were spotted on glow discharged, formvar-coated 300-mesh copper grids. Samples were left on grids for 2 min before blotting of excess liquid. Following blotting, grids were immediately stained with 1% phosphotungstic acid (PTA) and viewed with a Jeol JEM-1200EX transmission electron microscope at 80 kV.

### Vesicle preparation and FACS analysis

Vesicles were prepared from either JAWS II (ATCC) or *Anxa2*^*+/+*^ and *Anxa2*^*−/−*^ dendritic cells. After cell disruption by cavitation in 0.25 M sucrose in DPBS, and homogenization for 10 times with a Dounce homogenizer, the post-nuclear supernatant fraction was centrifuged at 2,000 × *g* for 15 min. The supernatant was then centrifuged at 100,000 × *g* for 1 h to eliminate residual fragments of the plasma membrane, ER, organelles and Golgi. The fraction of interest, which contained mostly vesicles, was harvested from the supernatant by pelleting at 300,000 × *g* for 1 h.

Vesicles were labelled with primary anti-annexin A2 (Clone A-15, Santa Cruz Biotechnology) and anti-Atg16L (Clone AB1, Sigma or Clone 1F12, MBL) antibodies, followed by the secondary goat anti-Alexa-488 and rabbit anti-Texas red-conjugated (Jackson ImmunoResearch) in staining buffer containing 220 mM KCl, 5 mM NaCl, 5 mM NaH_2_PO_4_, 0.5 mM MgCl_2_, pH 6.0. Antibody-stained vesicles were sorted on a Becton Dickinson FACS-Aria high-speed cell-sorting flow cytometer. Data acquisition and analysis were performed using the BD FACS Diva software.

### Proteomic analysis

FACS-sorted annexin A2^+^/Atg16L^+^ vesicles were lysed in 1% NP40 50 mM Tris/HCl and 150 mM NaCl for 1 h on ice. Protein concentration was determined by the Bradford method (Bio-Rad Laboratories). Proteins (15 μg) were resolved on a 10% SDS–PAGE, and protein bands visualized with the Silver Stain Kit for Mass Spectrometry (Pierce). Each lane was cut into slices, which were destained, and washed with 50 mM ammonium bicarbonate (NH_4_HCO_3_) and acetonitrile. Samples were then trypsin-digested, and peptides subjected to nano-LC-MS/MS sequencing on a LTQ-Orbitrap Velos HR mass spectrometer. MGF files were generated from the raw data files and searched against the SwissProt *Mus musculus* database using Mascot (version 2.1.04).

### Western blot analysis of Atg16L^+^ vesicles

Atg16L^+^ FACS-sorted vesicles were lysed in 1% NP40, 50 mM Tris/HCl and 150 mM NaCl lysis buffer. Protein concentration was determined by the Bradford method (Bio-Rad Laboratories). Total proteins (20 μg) were resolved on 4–15% gradient SDS–PAGE minigels (Bio-Rad). Total cell lysate and BMDC cytosol were run as positive controls. After transfer to nitrocellulose membranes and blocking in PBS with 0.1% Tween 20 and 5% non-fat milk, the membranes were incubated with anti-mouse annexin A2 (1:4,000, Clone C-10 Santa Cruz Biotechnology) or anti-mouse annexin A5 (1:200, Clone H-3, Santa Cruz Biotechnology), followed by horse radish peroxidase-conjugated secondary antibody (Jackson ImmunoResearch). Proteins were visualized by chemiluminescence (Supersignal, WB kit, Thermo Scientific).

### Vesicle fusion

Total cytosolic vesicles were prepared from *Anxa2*^*+/+*^ and *Anxa2*^*−/−*^ BMDC, as well as JAWS II cells by cavitation and ultracentrifugation, as described above. Vesicles were stained with Atg16L primary antibody; half of each vesicle preparation was stained subsequently with Alexa-488-conjugated mouse anti-rabbit IgG (Jackson ImmunoResearch), and the other half with TexasRed-conjugated goat anti-rabbit IgG (Invitrogen). In some experiments, Atg16L green and red FACS-sorted vesicles were incubated with increasing concentrations of Ca^2+^ (0, 2, 20 and 200 μM) for 40 min at 37 °C in 220 mM KCl, 5 mM NaCl, 5 mM NaH_2_PO_4_, 0.5 mM MgCl_2_, at pH 6.0. In other experiments, recombinant annexin A2 was added in the fusion buffer of *Anxa2*^*−/−*^ vesicles. In all experiments, single vesicles, double vesicles and fusion structures were enumerated in both green and red channels under an Olympus IX70 inverted microscope equipped with I.P. Lab Spectrum imaging software (Scanalytics Inc). A minimum of 300 vesicles and fusion events were counted in each experiments. For electron microscopy analyses, 10 μl of each sample was applied on Formvar-coated grid, and negatively stained with 1% PTA.

### Analysis of autophagic flux

Autophagic flux was determined by immunoblot analysis of cells treated with or without with 20 mM NH_4_Cl and 100 μM leupeptin to block lysosomal proteolysis^1^. The difference in LC3 levels between cells treated with or without the inhibitors was used to calculate autophagic flux, whereas the difference in LC3 levels at two times during the inhibition of proteolysis was used as an index of autophagosome formation. Autophagic flux was also measured upon transfection of the cells with the tandem reporter mCherry-GFP-LC3 (Addgene^38^). Cells were imaged 24 h after transfection and the number of mCherry-positive vesicles (autophagic vacuoles), mCherry and GFP-positive vesicles (autophagosomes), and mCherry-only-positive vesicles (autolysosomes) was calculated after thresholding, using the particle measure tool of the Image J software (NIH).

### Extraction of lipids and phosphoinositides

Extraction of lipids from the ATG16L^+^/*Anxa2*^*+/+*^ and from the ATG16L^+^/*Anxa2*^*−/−*^ autophagic vesicles was performed using a modification of the protocol developed by Honeyman^39^. Briefly, an acidic HCl solution was used to improve recovery of phosphoinositides, which tend to bind strongly to proteins during the standard extraction with chloroform/methanol solvent. Lyophilized vesicles were solubilized in 100 μl of H_2_O, to which 375 μl of chloroform/methanol/12N HCl (2/4/0.1, v/v) was added. After thorough mixing, 125 μl of chloroform was added, and the solution vortexed for 30 s followed by the addition of another 125 μl of H_2_O. After 10 min of centrifugation at 2,000 × *g*, the lower chloroform layer was removed and transferred to a glass tube for evaporation in a vacuum centrifuge. The lipid film was rapidly redissolved in 50 μl of 1:1:0.3 chloroform/methanol/water, and subjected to further analysis by thin layer chromatography (TLC) and nanoLC MS/MS.

### TLC lipid analysis

Lipid extracts from Atg16L^+^/*Anxa2*^*+/+*^ and Atg16L^+^/*Anxa2*^*−/−*^ vesicles were spotted on silica-coated plates, and developed in a closed jar with chloroform:methanol:water (65:25:1), supplemented with 1–2% phosphoric acid (H_3_PO_4_) to enable identification of major phospholipids and cholesterol. After the run, the TLC plate was air-dried and developed with iodine vapours. The dark-brown spots corresponding to the lipids of interest were identified using the corresponding lipid standards (PS=phosphatidylserine, PE=phosphatidylethanolamine, PC=phosphatidylcholine, cholesterol and the specific phophoinositides (PI=phosphatidyl inositol, PIP=phosphatidyl inositol monophosphate, PIP2=phosphatidyl inositol diphosphate and PIP3=phosphatidyl inositol triphosphate).

### MS analysis of lipid/phospholipid extracts

Lipid extract samples were diluted 1/10 in 1:1:0.3 (v/v/v) CHCl_3_:MeOH:H_2_O containing 25 mM piperidine^40^. Each sample (10 μl) was loaded into a 2-μm static nanospray emitter (PicoTips, New Objective). Using the static nanospray source at 1.0–1.4 kV, the sample typically lasted as a stable spray for 30 min. A high-resolution mass spectrometer (Orbitrap Velos, Thermo Scientific) was used in negative ionization mode for targeted and untargeted tandem MS (MS/MS), using a 1.5 *m/z* isolation width, and either collision-induced disassociation, higher collision disassociation or both at energies of 0 and 55%. Data collected at 0% provided the precursor ion *m/z* values obtained from the Orbitrap or from the Ion Trap. The untargeted approach utilized the Ion Map feature of the Orbitrap where profile MS/MS data (50–2,000 *m/z*) were collected from precursor ions from 200 to 2,000 *m/z*, at 2 *m/z* step size. Data were collected with the Xcalibur package (ThermoFinnigan).

### Lipidomics

The MS Analysis Tool provided by the Resources at the LIPID MAPS website (http://www.lipidmaps.org/resources/resources.html) was used to identify lipid species from MS/MS fragmentation profiles (Supplementary Data 1). Phospholipids (PI and PS) showing exact matches of their fragment ions with our experimental MS/MS data are shown in Fig. 3. The phospholipids are presented with the class abbreviation preceded by the *xx*:*y*, where *xx* is the total carbon atoms in the fatty acid chains and *y* is the number of double bonds.

### Analysis of intracellular protein degradation rates

Degradation of long-lived proteins in cultured cells was measured following 48-h labelling of cells with [^3^H] leucine (2 μCi ml^−l^) at 37 °C and pulse-chase experiments^41^. Cells were then extensively washed and placed in chase medium containing excess unlabelled leucine. Aliquots of the medium taken at different times were subjected to acid precipitation, and then filtered through a 0.22-μm to separate the filter-retained fraction (proteins) from the flow-through fraction (small peptides and amino acids). Proteolysis was calculated as the amount of acid-precipitable radioactivity (protein) transformed to acid soluble (amino acids) radioactivity during the incubation. MA-dependent degradation was inhibited using 10 mM 3-methyladenine, and lysosome-dependent degradation was inhibited using a mixture of 20 mM ammonium chloride and 100 μm leupeptine (N/L)^41^.

### Statistical methods

All numerical results are reported as mean±s.e.m. Statistical significance of the difference between experimental groups was analysed using the unpaired two-tailed Student’s *t*-test.

## Additional information

**How to cite this article:** Morozova, K. *et al*. Annexin A2 promotes phagophore assembly by enhancing Atg16^+^ vesicle biogenesis and homotypic fusion. *Nat. Commun.* 6:5856 doi: 10.1038/ncomms6856 (2015).

## Supplementary Material

Supplementary Figure and Supplementary Data Supplementary Figure 1

Supplementary Data 1 Library of identified glycerophospholipid species in the Annexin A2+/+ and -/- vesicles

## Figures and Tables

**Table 1:** Proteomic analysis of Annexin A2/Atg16L^+^ vesicles.

**Atg16L/Annexin A2+ vesicles**	**Function**	**Reference**
Actin	Vesicle trafficking	
Microtubule-actin cross-linking factor 1	Vesicle trafficking	
Twinfilin	Modulator of Actin polymerization	
WAS/WASL interacting protein (WIPF1)	Modulator of Actin polymerization	
Coronin-1	Autophagosome formation	42
Cortactin	Autophagosome–lysosomal fusion	43
Dynactin	Autophagosome movement	44
Cofilin 1	Modulator of Actin polymerization	
AP-2 (beta and mu subunits)	Atg16L interaction	7
Clathrin	Atg16L interaction	7
Synaptotagmin II	Endocytosis	
Synaptojanin 2	PI(4,5)P2 hydrolysis	
Endophilin A3	Endocytosis; vesicular transport	
Annexin A2	Lipid segregation (PS and PIs)	
Protein S100A11	Annexin A2-binding partner	
ASAP2	ARF6 regulator	
Vesicle-fusing ATPase	Vesicle trafficking	
ARF6	PIP2 formation via PIP5K; PLD Inhibition	6
Girdin	Autophagy regulator	30
VAMP 7	SNARE	7
VAMP 3	SNARE	5
VtiB	SNARE	7
Ubiquitin	Binding to Atg16L	45
Galectin 3	Endocytosis	
Aurora A kinase	Autophagy regulators	31
Vps 13c	Endocytosis; Regulation PI phosphates	
Hrb	VAMP 7 adaptor	7

MA, macroautophagy; PI, phosphatidylinositol; PS, phosphatidylserine.

Proteins previously reported to be associated with MA are referenced.

## References

[b1] Klionsky D. J. . Guidelines for the use and interpretation of assays for monitoring autophagy. Autophagy 8, 445–544 (2012). 22966490 10.4161/auto.19496 PMC3404883

[b2] Ravikumar B. . Mammalian macroautophagy at a glance. J. Cell Sci. 122, 1707–1711 (2009). 19461070 10.1242/jcs.031773 PMC2684830

[b3] Ohsumi Y. & Mizushima N. Two ubiquitin-like conjugation systems essential for autophagy. Semin. Cell Dev. Biol. 15, 231–236 (2004). 15209383 10.1016/j.semcdb.2003.12.004

[b4] Puri C., Renna M., Bento C. F., Moreau K. & Rubinsztein D. C. Diverse autophagosome membrane sources coalesce in recycling endosomes. Cell 154, 1285–1299 (2013). 24034251 10.1016/j.cell.2013.08.044 PMC3791395

[b5] Puri C., Renna M., Bento C. F., Moreau K. & Rubinsztein D. C. ATG16L1 meets ATG9 in recycling endosomes: additional roles for the plasma membrane and endocytosis in autophagosome biogenesis. Autophagy 10, 182–184 (2014). 24257061 10.4161/auto.27174 PMC4389876

[b6] Moreau K., Renna M. & Rubinsztein D. C. Connections between SNAREs and autophagy. Trends Biochem. Sci. 38, 57–63 (2013). 23306003 10.1016/j.tibs.2012.11.004

[b7] Moreau K., Ravikumar B., Renna M., Puri C. & Rubinsztein D. C. Autophagosome precursor maturation requires homotypic fusion. Cell 146, 303–317 (2011). 21784250 10.1016/j.cell.2011.06.023 PMC3171170

[b8] Walker S., Chandra P., Manifava M., Axe E. & Ktistakis N. T. Making autophagosomes: localized synthesis of phosphatidylinositol 3-phosphate holds the clue. Autophagy 4, 1093–1096 (2008). 18927492 10.4161/auto.7141

[b9] Axe E. L. . Autophagosome formation from membrane compartments enriched in phosphatidylinositol 3-phosphate and dynamically connected to the endoplasmic reticulum. J. Cell Biol. 182, 685–701 (2008). 18725538 10.1083/jcb.200803137 PMC2518708

[b10] Hamasaki M. . Autophagosomes form at ER-mitochondria contact sites. Nature 495, 389–393 (2013). 23455425 10.1038/nature11910

[b11] Hamasaki M., Shibutani S. T. & Yoshimori T. Up-to-date membrane biogenesis in the autophagosome formation. Curr. Opin. Cell Biol. 25, 455–460 (2013). 23578367 10.1016/j.ceb.2013.03.004

[b12] Hamasaki M. & Ohsumi Y. [Involvement of the secretory pathway in the autophagosome formation]. Tanpakushitsu. Kakusan. Koso. 51, 1469–1473 (2006). 16922421

[b13] Hailey D. W. . Mitochondria supply membranes for autophagosome biogenesis during starvation. Cell 141, 656–667 (2010). 20478256 10.1016/j.cell.2010.04.009 PMC3059894

[b14] Law A. L. . Annexin A2 regulates phagocytosis of photoreceptor outer segments in the mouse retina. Mol. Biol. Cell 20, 3896–3904 (2009). 19587120 10.1091/mbc.E08-12-1204 PMC2735488

[b15] Hedhli N. . The annexin A2/S100A10 system in health and disease: emerging paradigms. J. Biomed. Biotechnol. 2012, 406273 (2012). 23193360 10.1155/2012/406273 PMC3496855

[b16] Moss S. E. & Morgan R. O. The annexins. Genome Biol. 5, 219 (2004). 15059252 10.1186/gb-2004-5-4-219 PMC395778

[b17] Gerke V. & Moss S. E. Annexins: from structure to function. Physiol. Rev. 82, 331–371 (2002). 11917092 10.1152/physrev.00030.2001

[b18] Donnelly S. R. & Moss S. E. Annexins in the secretory pathway. Cell. Mol. Life Sci. 53, 533–538 (1997). 9230932 10.1007/s000180050068 PMC11147375

[b19] Grieve A. G., Moss S. E. & Hayes M. J. Annexin A2 at the interface of actin and membrane dynamics: a focus on its roles in endocytosis and cell polarization. Int. J. Cell Biol. 2012, 852430 (2012). 22505935 10.1155/2012/852430 PMC3296266

[b20] Hayes M. J. . Annexin 2 binding to phosphatidylinositol 4,5-bisphosphate on endocytic vesicles is regulated by the stress response pathway. J. Biol. Chem. 279, 14157–14164 (2004). 14734570 10.1074/jbc.M313025200 PMC1351152

[b21] Gruenberg J. & Stenmark H. The biogenesis of multivesicular endosomes. *Nature reviews*. Nat. Rev. Mol. Cell Biol. 5, 317–323 (2004). 15071556 10.1038/nrm1360

[b22] Mayran N., Parton R. G. & Gruenberg J. Annexin II regulates multivesicular endosome biogenesis in the degradation pathway of animal cells. EMBO J. 22, 3242–3253 (2003). 12839987 10.1093/emboj/cdg321 PMC165635

[b23] Hayes M. J. . Annexin A2 at the interface between F-actin and membranes enriched in phosphatidylinositol 4,5,-bisphosphate. Biochim. Biophys. Acta 1793, 1086–1095 (2009). 19022301 10.1016/j.bbamcr.2008.10.007 PMC7611824

[b24] Hayes M. J., Shao D., Bailly M. & Moss S. E. Regulation of actin dynamics by annexin 2. EMBO J. 25, 1816–1826 (2006). 16601677 10.1038/sj.emboj.7601078 PMC1456940

[b25] Merrifield C. J. . Annexin 2 has an essential role in actin-based macropinocytic rocketing. Curr. Biol. 11, 1136–1141 (2001). 11509239 10.1016/s0960-9822(01)00321-9

[b26] Scharf B. . Annexin A2 binds to endosomes following organelle destabilization by particulate wear debris. Nat. Commun. 3, 755 (2012). 22453828 10.1038/ncomms1754 PMC3606553

[b27] Randow F. & Munz C. Autophagy in the regulation of pathogen replication and adaptive immunity. Trends Immunol. 33, 475–487 (2012). 22796170 10.1016/j.it.2012.06.003 PMC3461100

[b28] Gannage M. & Munz C. Autophagy in MHC class II presentation of endogenous antigens. Curr. Top. Microbiol. Immunol. 335, 123–140 (2009). 19802563 10.1007/978-3-642-00302-8_6

[b29] Singh S. B., Davis A. S., Taylor G. A. & Deretic V. Human IRGM induces autophagy to eliminate intracellular mycobacteria. Science 313, 1438–1441 (2006). 16888103 10.1126/science.1129577

[b30] Garcia-Marcos M., Ear J., Farquhar M. G. & Ghosh P. A GDI (AGS3) and a GEF (GIV) regulate autophagy by balancing G protein activity and growth factor signals. Mol. Biol. Cell 22, 673–686 (2011). 21209316 10.1091/mbc.E10-08-0738 PMC3046063

[b31] Zou Z. . Aurora kinase A inhibition-induced autophagy triggers drug resistance in breast cancer cells. Autophagy 8, 1798–1810 (2012). 23026799 10.4161/auto.22110 PMC3541289

[b32] Park J. S. & Neiman A. M. VPS13 regulates membrane morphogenesis during sporulation in *Saccharomyces cerevisiae*. J. Cell Sci. 125, 3004–3011 (2012). 22442115 10.1242/jcs.105114 PMC3434806

[b33] Samaranayake H. S., Cowan A. E. & Klobutcher L. A. Vacuolar protein sorting protein 13A, TtVPS13A, localizes to the tetrahymena thermophila phagosome membrane and is required for efficient phagocytosis. Eukaryot. Cell 10, 1207–1218 (2011). 21764909 10.1128/EC.05089-11 PMC3187053

[b34] Posor Y. . Spatiotemporal control of endocytosis by phosphatidylinositol-3,4-bisphosphate. Nature 499, 233–237 (2013). 23823722 10.1038/nature12360

[b35] Lee S. J., Feldman R. & O'Farrell P. H. An RNA interference screen identifies a novel regulator of target of rapamycin that mediates hypoxia suppression of translation in Drosophila S2 cells. Mol. Biol. Cell 19, 4051–4061 (2008). 18653470 10.1091/mbc.E08-03-0265 PMC2555944

[b36] Simonsen A., Wurmser A. E., Emr S. D. & Stenmark H. The role of phosphoinositides in membrane transport. Curr. Opin. Cell Biol. 13, 485–492 (2001). 11454456 10.1016/s0955-0674(00)00240-4

[b37] Menke M., Gerke V. & Steinem C. Phosphatidylserine membrane domain clustering induced by annexin A2/S100A10 heterotetramer. Biochemistry 44, 15296–15303 (2005). 16285733 10.1021/bi051585i

[b38] Pampliega O. . Functional interaction between autophagy and ciliogenesis. Nature 502, 194–200 (2013). 24089209 10.1038/nature12639 PMC3896125

[b39] Honeyman T. W., Strohsnitter W., Scheid C. R. & Schimmel R. J. Phosphatidic acid and phosphatidylinositol labelling in adipose tissue. Relationship to the metabolic effects of insulin and insulin-like agents. Biochem. J. 212, 489–498 (1983). 6411068 10.1042/bj2120489 PMC1152072

[b40] Milne S. B., Ivanova P. T., DeCamp D., Hsueh R. C. & Brown H. A. A targeted mass spectrometric analysis of phosphatidylinositol phosphate species. J. Lipid Res. 46, 1796–1802 (2005). 15897608 10.1194/jlr.D500010-JLR200

[b41] Kaushik S., Massey A. C., Mizushima N. & Cuervo A. M. Constitutive activation of chaperone-mediated autophagy in cells with impaired macroautophagy. Mol. Biol. Cell 19, 2179–2192 (2008). 18337468 10.1091/mbc.E07-11-1155 PMC2366850

[b42] Seto S., Tsujimura K. & Koide Y. Coronin-1 inhibits autophagosome formation around Mycobacterium tuberculosis-containing phagosomes and assists mycobacterial survival in macrophages. Cell Microbiol. 5, 710–727 (2012). 10.1111/j.1462-5822.2012.01754.x 22256790

[b43] Lee J. Y. . HDAC6 controls autophagosome maturation essential for ubiquitin-selective quality-control autophagy. EMBO J. 29, 969–980 (2010). 20075865 10.1038/emboj.2009.405 PMC2837169

[b44] Kimura S., Noda T. & Yoshimori T. Dynein-dependent movement of autophagosomes mediates efficient encounters with lysosomes. Cell Struct. Funct. 33, 109–122 (2008). 18388399 10.1247/csf.08005

[b45] Fujita N. . Recruitment of the autophagic machinery to endosomes during infection is mediated by ubiquitin. J. Cell Biol. 203, 115–128 (2013). 24100292 10.1083/jcb.201304188 PMC3798248

