# Supplementary material for: Annexin A2 promotes phagophore assembly by enhancing Atg16L+ vesicle biogenesis and homotypic fusion
Source: Nat Commun. 2015 Jan 19;6:5856. doi: 10.1038/ncomms6856 (PMC4299943; doi:10.1038/ncomms6856)
Supplement: Supplementary Figure and Supplementary Data — Supplementary Figure 1 [file ncomms6856-s1.pdf]

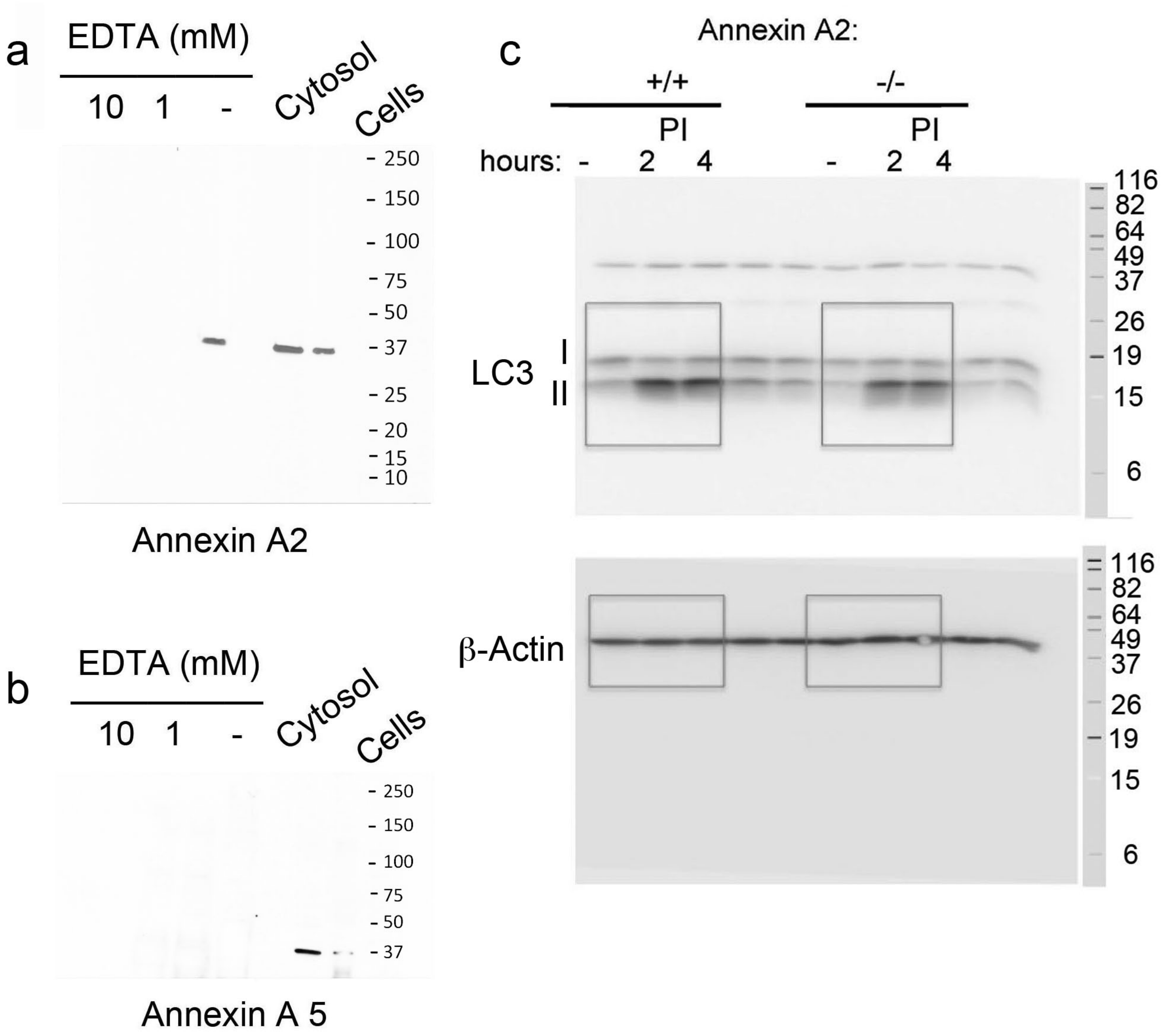

Supplementary Figure 1. Western blots reported in Figures 1 and 5
a) western blot for Annexin A2 reported in Figure 1g. **b)** western blot for Annexin A5 reported in Figure 1g **c)** western blot for LC3 and beta-actin reported in Figure 5d.
